# Characterization of Fungi Communities in Organic Soybean Seeds Using DNA Sequencing: Effects of Cultivar and Location

**DOI:** 10.3390/pathogens15020239

**Published:** 2026-02-23

**Authors:** Hanna Olszak-Przybyś, Marcin Przybyś, Jolanta Bojarszczuk, Jerzy Księżak

**Affiliations:** 1 Department of Biotechnology and Plant Breeding, Institute of Soil Science and Plant Cultivation—State Research Institute, ul. Czartoryskich 8, 24-100 Puławy, Poland; mprzybys@iung.pulawy.pl; 2 Department of Crops and Yield Quality, Institute of Soil Science and Plant Cultivation—State Research Institute, ul. Czartoryskich 8, 24-100 Puławy, Poland; jbojarszczuk@iung.pulawy.pl (J.B.); jksiezak@iung.pulawy.pl (J.K.)

**Keywords:** fungal colonization, seed-borne fungi, fungal diversity, seed quality

## Abstract

Organic soybean seeds are susceptible to colonization by numerous fungal pathogens, which can reduce their germination capacity and nutritional quality. This study evaluated fungi transmitted by seeds and their effects on selected seed quality parameters, as well as the influence of variety, location and growing season. In total, 471 fungal isolates belonging to 24 genera and 37 species were obtained from three soybean varieties (Erica, Es Commandor and Cerez PZO) cultivated at two locations during the 2022–2023 seasons. All obtained isolates were identified based on *ITS* sequencing, and *Fusarium* isolates were further characterized to the species level using *TEF* and *RPB2* markers. *Fusarium* spp. was the most frequently isolated genus, accounting for 35.7% of all isolates, followed by *Alternaria* spp. (15.9%) and *Aspergillus* spp. (11.9%). Fungal frequency and species diversity differed significantly between years. Seed germination capacity was significantly lower in 2023 than in 2022 and coincided with higher fungal colonization, lower spring temperatures, and increased rainfall. A significant negative Pearson’s correlation (r = −0.58, *p* < 0.05) was found between fungi abundance and seed oil content, indicating a direct impact of fungal colonization on nutritional quality. These results highlight the role of environmental conditions in seed-borne pathogen communities and the need for monitoring and seed health management to ensure soybean seed quality.

## 1. Introduction

Soybean (*Glycine max* L. Merr.) is one of the world’s most important crops, due to the high nutritional quality of its seeds. These seeds contain approximately 35–40% protein with a well-balanced amino acid profile, making soybean a key source of protein in human and animal nutrition [1,2,3,4]. In addition, soybean seeds are rich in oil containing high levels of unsaturated fatty acids, such as linoleic and α-linolenic acids. Therefore, soybean is an important raw material for the food industry [2,3,5].

Due to its high nutritional and economic value, soybean plays an important role in sustainable agriculture, including organic farming systems. However, organic farming has certain limitations, especially with regard to the use of synthetic plant protection products. Their absence can lead to an increase in problems associated with fungal pathogens transmitted by seeds. These fungi pose a serious threat to organic soybean cultivation as they colonize seed material, reduce germination capacity and negatively affect seed quality by impairing their chemical composition and viability [6,7,8,9,10].

Global soybean production remains concentrated in a few major countries, mainly Brazil, the United States, and Argentina. Together, these countries account for nearly 80% of global soybean output. However, soybean cultivation is gradually expanding to other regions, including Central and Eastern Europe. This trend is driven by rising demand for plant-based protein and the need to diversify cropping systems [11,12]. In Poland, the area of soybean cultivation has increased rapidly in recent years. This growth is due to a number of factors, including growing demand for domestic protein sources to reduce dependence on imported soybean, the development and availability of varieties adapted to local climatic conditions, and financial support for protein crops. Favorable agronomic conditions and growing awareness of the benefits of crop diversification in sustainable agriculture have further contributed to this growth. In 2023, soybean was cultivated on approximately 44.600 hectares. In 2024, the area cultivation increased to about 79.800 hectares, and in 2025 it reached 98.100 hectares. These data confirm a strong upward trend in soybean production in Poland [13].

Using high-quality seed material is essential for soybean cultivation in sustainable agricultural systems. Seeds designated for sowing must have high germination capacity, be healthy, and have a stable chemical composition. Seed and soil management have a major impact on seed quality. Previous studies have shown that suboptimal seed health or low viability causes delayed and uneven emergence. This reduces yield potential, especially in low-input organic systems where chemical treatments and synthetic fertilizers are limited [14]. Comparative studies of organic and conventional soybean cultivation demonstrated that seed composition, such as protein, amino acid and fat content, can vary with the cultivation method and environmental conditions [15]. In the context of organic farming, using high-quality seeds material is crucial. Such seeds reduce losses caused by pathogens and help achieve stable yields. In organic soybean production, it is particularly important that seeds are free from plant pathogens, which can affect germination and seedling vigor. Therefore, the use of certified or qualified seed material, which meets strict health and quality standards, is essential to ensure successful cultivation and maintain the sustainability of organic cropping systems [16].

Environmental conditions, especially humidity and temperature, strongly affect fungal on soybean seeds [17,18]. Fungi such as *Alternaria* and *Fusarium* need favorable conditions to infect seeds. Their occurrence usually increases during long rainy periods in the seed maturation and harvest periods [19]. High air and seed humidity promotes spore germination and fungal growth, causing seed infection and deterioration in quality [20]. Temperature also affects fungal growth. Many fungi show optimal growth at moderate to high temperatures, where colonization and seed damage occur more rapidly [21,22]. Warm and wet weather during seed maturation and harvest increases infection risk. Effective seed health management should consider these factors when determining harvest timing and storage to minimize pathogen growth and preserve seed viability.

One of the major threats to soybean seeds quality is fungal infection. This can occur before and after harvest. More than 150 fungal species have been reported on soybean seeds. The most common seed-borne pathogens belong to the genera *Fusarium*, *Alternaria*, *Aspergillus*, *Chaetomium*, *Cercospora*, *Ascochyta*, *Diaporthe*, and *Cladosporium* [6,23,24]. These pathogens can reduce seed germination, weaken seedling emergence, and cause root diseases or seedling death. Fungal infections of pods also affect seed quality and quantity, reducing yields and commercial value. Infected seeds are a major source of pathogen spread, promoting disease transmission locally and over long distances [6,25]. Fungal colonization may further impair the organoleptic quality of seeds and reduce oil content, thereby negatively affecting the nutritional and technological value of soybean seeds [9].

Species of the genus *Fusarium*, due to their high frequent occurrence and strong pathogenicity, pose a serious threat to soybean cultivation and seed quality. Soybean seeds infected with *Fusarium* often have reduced germination capacity. Pathogenic species such as *F. fujikuroi*, *F. equiseti*, *F. graminearum*, and *F. proliferatum* can markedly reduce emergence and seedling vigor compared to healthy seeds. This clearly demonstrates the strong negative impact of *Fusarium* on seed quality [20,26,27]. In addition to reducing germination, *Fusarium* infections can also impair the chemical composition of seeds, including lowering total protein content [9]. Many studies on fungal communities in soybean have shown that *Fusarium* is the dominant genus and the main pathogen transmitted by the seeds of this plant [9,26,28].

*Alternaria* infections also reduce seed germination capacity and cause visible changes such as seed shrinkage and brow discoloration. In severely infected seeds, the pathogen’s mycelium can completely destroy the endosperm [7,29]. Similarly, fungi of the genus *Aspergillus* can impair seed quality. They damage nutrients and produce mycotoxin that threaten food and feed safety [8,10]. Therefore, effective control of these fungal infections is important for maintaining high-quality soybean seeds.

Accurate identification of fungi on soybean seeds is essential to assess phytosanitary risk. Correct detection and identification of fungal species help understand their impact on plant health. It is also important for developing management strategies and preventing pathogens spread through seeds [29]. In this context, sequencing the Internal Transcribed Spacer (*ITS*) region is especially useful. The *ITS* region is widely recognized as a universal DNA barcode for fungi. It allows precise differentiation of closely related species. *ITS* sequencing is highly effective and widely used in fungal diagnostics and systematics [30,31,32,33].

Numerous studies have documented the presence of various fungal taxa on the surface and inside soybean seeds, highlighting their importance for seed and crop health. Escamilla et al. (2019) [23] identified several types of seed-borne fungi, including *Alternaria*, *Fusarium*, and others, on commercial soybean seeds. Hartman et al. [6] provide an overview of the major pathogens and pests of soybeans, pointing out that fungi are an important component of seed-associated microflora and can cause serious diseases, including seed decay, seedling blight, purple seed stain, and stem blight. Roy et al. [24] reviewed the microflora present on soybean seeds and showed that both field and storage fungi can colonize seeds and potentially deteriorate their quality. Recently, studies by Olszak-Przybyś and Korbecka-Glinka [9] on soybean seeds from southern Poland revealed a high diversity of seed-borne fungi, with *Aspergillus*, *Alternaria* and *Fusarium* being the most frequently isolated genera. The authors showed that *Fusarium* reduced seed protein content, whereas *Aspergillus* and *Penicillium* decreased oil content and increased the level of free fatty acids. Overall, these studies indicate that soybean seeds provide a habitat for many fungal species, highlighting the need for comprehensive community analyses in different cropping systems.

However, a few studies have specifically addressed seed from organic farming systems. In this study, it was combined microbiological assessment with the analysis of selected chemical parameters. It was also evaluated the germination capacity. As part of a comprehensive characterization of organic soybeans from three cultivars grown in two locations during the 2022–2023 seasons, the influence of environmental conditions, such as temperature and humidity, on seed germination was assessed.

## 2. Materials and Methods

### 2.1. Plant Material and Experimental Fields

The experimental material consisted of seeds from three soybean cultivars: Es Commandor (breeder: LIDEA, Lescar, France), Cerez PZO (IGP, Ismaning, Germany), and Erica (DANKO, Kościan, Poland), representing different maturity groups according to the COBORU (The Research Centre for Cultivar Testing) classification (Table 1) [34]. Seed samples were obtained from organic variety trials conducted by COBORU during the 2022 and 2023 growing seasons at two experimental locations in Poland: Przecław (50°11′39″ N 21°28′47″ E; Podkarpackie Province) and Tarnów (50°34′43″ N 16°47′20″ E; Dolnośląskie Province). The experimental sites are located in a temperate continental climate zone. For each cultivar, year, and location, seeds harvested from the four field replications were pooled to obtain one composite sample, which was used for further analyses. All samples were collected and prepared in accordance with the International Seed Testing Association (ISTA) sampling methodology and used for further analyses [35]. Meteorological conditions, including average monthly air temperatures and total monthly precipitation during the 2022 and 2023 seasons, were recorded at each experimental field by COBORU. Detailed weather data are presented in Table 2.

Field experiments were conducted using a randomized block design with four replications for each of the three cultivars. In Tarnów, the experiment was carried out on podzolic soil classified as a very good wheat complex (soil class IIIa), whereas in Przecław the trial was conducted on medium loamy soil belonging to the very good wheat complex (soil class II). The forecrops in Tarnów were spring wheat in 2020 and spring barley in 2021, while in Przecław oat and spring barley were used, respectively. Sowing was performed at a depth of 3–4 cm, and all cultivars were sown within the first ten days of May. Before sowing, seeds were inoculated with a commercial peat-based inoculant (Nitragina; IUNG-PIB, Puławy, Poland) containing *Bradyrhizobium japonicum* strains specific to soybean. No chemical treatments or mineral fertilization was applied during the experiment.

### 2.2. Germination Test

Seed germination tests were performed according to the International Seed Testing Association (ISTA) protocols [36]. Three replicates of 100 seeds (300 total) were used for each soybean cultivar. The cultivars were evaluated at two locations over two growing seasons, resulting in 1.200 seeds per cultivar and a total of 3.600 seeds for the study. Seeds were placed between moist filter paper rolls, enclosed in plastic bags, and incubated in a climatic chambers (MC-1750, Snijders, Tilburg, The Netherlands). The conditions were 30 °C for 8 h (light) and 20 °C for 16 h (darkness), at 90% relative humidity. After eight days of incubation, the number of germinated seeds was recorded. Germination percentage was calculated for each replicate, with a distinction made between normal and abnormal seedlings, as defined by ISTA rules [36].

### 2.3. Isolation of Seed-Borne Fungi and DNA Amplicon Sequencing

Fungal isolation was performed using 100 seeds from each seed lot, arranged in four replicates of 25 seeds. The seeds were surface-disinfected with a 1.4% sodium hypochlorite solution (Chempur, Piekary Śląskie, Poland) for one minute. They were then rinsed three times with sterile distilled water and air-dried on sterile filter paper. The disinfected seeds were placed on potato dextrose agar (PDA; Difco™, Sparks, MD, USA) with 2.5 mg·L^−1^ tetracycline hydrochloride (Duchefa Biochemie, Haarlem, The Netherlands). Five Petri dishes with five seeds in each dish were used in every replication. The plates were incubated at 20 °C for 14 days under a 12 h photoperiod (8 h light/16 h dark). The emerging fungal colonies were subcultured by transferring fragments of actively growing mycelium to a fresh PDA plate. After repeated transfer of the edge mycelium using a needle, cultures were obtained, which then used for molecular identification. Genomic DNA was extracted from pure cultures using a modified CTAB method [37]. The extraction buffer contained: 3% (*w*/*v*) CTAB (AppliChem, Darmstadt, Germany), 100 mM Tris-base (Thermo Scientific, Rockford, IL, USA), 20 mM EDTA (Invitrogen, Grand Island, NY, USA), and 1.4 M NaCl (Avantor, Gliwice, Poland), adjusted to pH 8.0. The concentration and purity of the extracted DNA were evaluated spectrophotometrically using a NanoDrop 2000 spectrophotometer (Thermo Scientific, Wilmington, DE, USA). DNA extracts were normalized to a concentration of 10 ng/µL. All isolates were amplified and sequenced in the *ITS* region, a widely accepted fungal barcode. Additionally, *Fusarium* isolates were identified at the species level by sequencing highly informative genomic regions: the translation elongation factor (TEF1) and RNA polymerase second largest subunit (RPB2). The initial PCR reactions for all regions studied (*ITS*, *TEF*, *RPB2*) was performed using a two-step PCR method. In the first step, primers with Illumina compatible adapters were applied to enable amplification of the target regions (Table 3). PCR reactions were carried out using KAPA HiFi Hot Start Ready Mix (2×) (Roche, Kapa Biosystems, Cape Town, RPA) under standard amplification conditions. In the second step, the obtained amplicons were labeled with dual Illumina indices using the Nextera XT Index Kit v2 (Illumina, San Diego, CA, USA). After each PCR reaction, the products were purified using Agencourt AMPure XP magnetic beads (Beckman Coulter, Indianapolis, IN, USA). Fragment length validation and library quality assessment were performed using a 2100 Bioanalyzer (Agilent Technologies, Santa Clara, CA, USA) with a DNA 1000 chip (Agilent Technologies, Santa Clara, CA, USA). Quantitative evaluation of the obtained DNA libraries was carried out using the QuantiFluor^®^ dsDNA System (Promega, Madison, WI, USA) and a Quantus™ fluorometer (Promega, Madison, WI, USA). The concentrations of all libraries were normalized, after which individual libraries were pooled and subjected to paired-end sequencing (2 × 300 bp) on an Illumina MiSeq platform (Illumina, San Diego, CA, USA). To increase sequence diversity and improve sequencing quality, 15% PhiX control was added to the sequencing run.

After sequencing, FASTQ files containing DNA sequences were obtained. Analyses were performed using the QIIME2 (ver. 2025.10) software package [40]. In the first step, the files were demultiplexed, allowing assignment of individual sequences to their respective samples. Sequence quality and sequencing depth were then assessed. Based on these results, the sequence lengths used in subsequent analyses were determined. Data denoising was performed using the DADA2 denoise-paired tool. The final step of the analysis involved taxonomic assignment, which was conducted using the feature-classifier classify-sklearn tool with default parameter settings and the latest UNITE fungal *ITS* reference database (ver. 10_19_02_2025) (https://unite.ut.ee accessed on 12 January 2026). Representative sequences of each fungal species detected in the analyzed seeds were deposited in the GenBank database (accession no. PX875849—PX875891) and additionally were compared with similar sequences in the NCBI database using the BLAST nucleotide search programme (ver. 2.17.0+; http://blast.ncbi.nlm.nih.gov accessed on 13 January 2026) (Table S1). Additionally, representative sequences of the *RPB2* and *TEF1* gene regions of each identified *Fusarium* species were deposited in the GenBank database. These sequences were also subjected to BLASTn analysis against the NCBI nucleotide database (http://blast.ncbi.nlm.nih.gov) accessed on 12 February 2026 (Table S2).

### 2.4. Seed Quality Analysis

Seed quality was evaluated by measuring total nitrogen, crude protein, and fat content were expressed on a dry weight basis. Thousand seed weight (TSW) was also determined for each sample. All chemical analyses were conducted at the Central Chemical Laboratory (GLACH) of the Institute of Soil Science and Plant Cultivation—State Research Institute (IUNG), Puławy, Poland. Total nitrogen was measured using the Kjeldahl method, involving digestion in concentrated sulfuric acid followed by spectrophotometric flow analysis [41]. Crude protein (CP) content was calculated using the following equation:

CP = N × 6.25

where:

CP = crude protein content (%)
N = total nitrogen content (%)
6.25 = conversion factor for plant proteins

Fat content was assessed using the Soxhlet extraction method with carbon tetrachloride as the solvent [42]. All tested samples were analysed in three analytical replications.

### 2.5. Statistical Analysis

All data were prepared using Microsoft Excel (Microsoft Corp., Redmond, WA, USA), and statistical analyses were performed using Statistica, version 13.3 (TIBCO Software Inc., Palo Alto, CA, USA). Based on the germination results, arithmetic means and standard deviations were calculated for each seed lot. The effect of experimental field location, soybean cultivar, and harvest year on germination percentage was evaluated using analysis of variance (ANOVA). Tukey’s HSD test was used for pairwise comparisons at *p =* 0.05. Pearson’s correlation coefficients were calculated to assess relationships between selected soybean seed traits (total oil, protein, nitrogen, moisture, and germination) and the number of fungal isolates. Only statistically significant correlations (*p* < 0.05) were considered.

## 3. Results

### 3.1. Seed Germination and Quality by Year and Location

A post hoc analysis using Tukey’s HSD test (α = 0.05) revealed significant differences in seed germination capacity depending on the harvest year (Table 4). The lowest germination was observed in 2023 for Cerez PZO (43.3%) and Es Commandor (43.7%) varieties grown in Tarnów and for Es Commandor variety cultivated in Przecław (45.7%). These values differed significantly from all other groups. In 2023, cultivar Erica showed higher germination, reaching 64.0% in Tarnów and 68.7% in Przecław. However, these values were still significantly lower than those recorded in 2022.

In 2022, seed germination capacity was high for almost all cultivars and locations, exceeding 95%, with the exception of Erica grown in Tarnów, which showed 81.7% germination and was statistically different from other treatments. The highest value was recorded for Cerez PZO variety grown in Przecław (99.3%). No significant differences were found among these cultivars. Only Erica variety grown in Tarnów showed a lower germination capacity (81.7%) (Table 4). The results obtained indicate that harvest year had a significant impact on seed germination capacity.

The lower germination observed in 2023 may be related to weather conditions during the growing season. At both locations, average temperatures in May and June 2023 were much lower than in 2022. This may have slowed plant growth and development. In Przecław, the total precipitation in June 2023 was markedly higher (80.0 mm) compared with 2022 (21.3 mm) (Table 2). High rainfall increases air humidity and promotes fungal development, which can reduce seed germination. Despite lower germination, the thousand seed weight (TSW) was generally higher in 2023 compared with 2022.

### 3.2. Diversity of Fungal Species in Soybean Seeds

A total of 471 fungal isolates were obtained from the seed samples tested. They represented 24 genera and 37 species. The most abundant genus was *Fusarium* spp., accounting for 35.7% of all isolates. *Alternaria* spp. (15.9%), *Aspergillus* spp. (11.9%), *Boeremia* spp. (8.7%) and *Diaporthe* spp. (8.3%) were less common. The remaining genera were rare and usually occurred at frequency of less than 5%. Sporadically occurring taxa (0.2%) included *Pseudeurotium* sp., *Apiospora* sp., *Nigrospora* sp., *Stemphylium* sp., and *Syncephalastrum* sp. (Table 5).

Most of the identified fungi belonged to the genus *Fusarium*, which was confirmed by sequencing the *ITS* region, as well as the *TEF* and *RPB2* regions. Five species were distinguished within this genus. The most numerous was *F. equiseti*, with 65 isolates. It accounted for 13.8% of all isolates and 38.7% of *Fusarium* isolates. *F. culmorum* (27 isolates) and *F. flagelliforme* (26 isolates) were also frequently found. *F. avenaceum* and *F. graminearum* were less numerous, with 17 and 12 isolates, respectively. Lower numbers of isolate were recorded for *F. sporotrichioides* (10 isolates) and *F. lateritium* (7 isolates), whereas *F. acuminatum* was the least frequently detected species, represented by only four isolates (Table 6).

The second most abundant genus was *Alternaria.* It was mainly represented by *A. prunicola*, with 72 isolates (15.3% of all fungal isolates). *A. alternata* was rare and accounted for only 0.4% of the total isolates (Table 6).

*Aspergillus* was also frequently isolated, with 56 isolates (11.9% of all fungi). *ITS* sequencing allowed seven species within this genus (Table 6). The most common species were *A. tubingensis* (19 isolates), *A. flavus* (18 isolates), and *A. luchuensis* (9 isolates). The remaining species were represented by four or fewer isolates.

Only one species *Boeremia exigua*, was identified within the genus *Boeremia*, accounting 0.4% of all isolates. In the case of the remaining 39 isolates, *ITS* sequencing did not allow for clear identification at the species level.

The genus *Diaporthe* was represented by three species. The prevalent was *D. cotoneastri*, representing 46.2% of *Diaporthe* isolates. *D. eres* constituted 35.9% and *D. subclavata* 17.9%. (Table 6).

### 3.3. Fungal Community Structure and Correlations with Soybean Seed Traits

**Figure 1:**
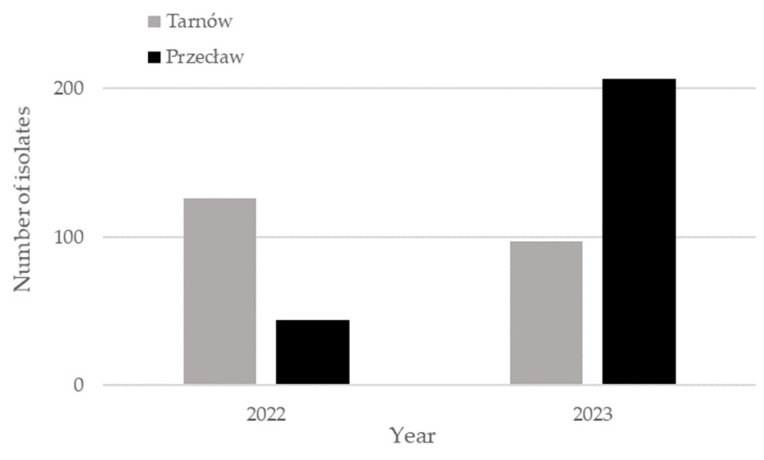
Sum of fungal isolates obtained from soybean seeds in 2022 and 2023.

Significant differences in the number and diversity of fungal were observed between individual years and locations. In 2022, a total of 170 isolates were obtained, including 44 from Przecław and 126 from Tarnów (Figure 1). In 2023, the total number of isolates increased markedly to 301, of which 207 came from Przecław and 97 from Tarnów. The higher number of isolates obtained in 2023, especially in Przecław, may be linked to lower spring temperatures and higher air humidity in the summer, which favoured the development of fungi.

**Figure 2:**
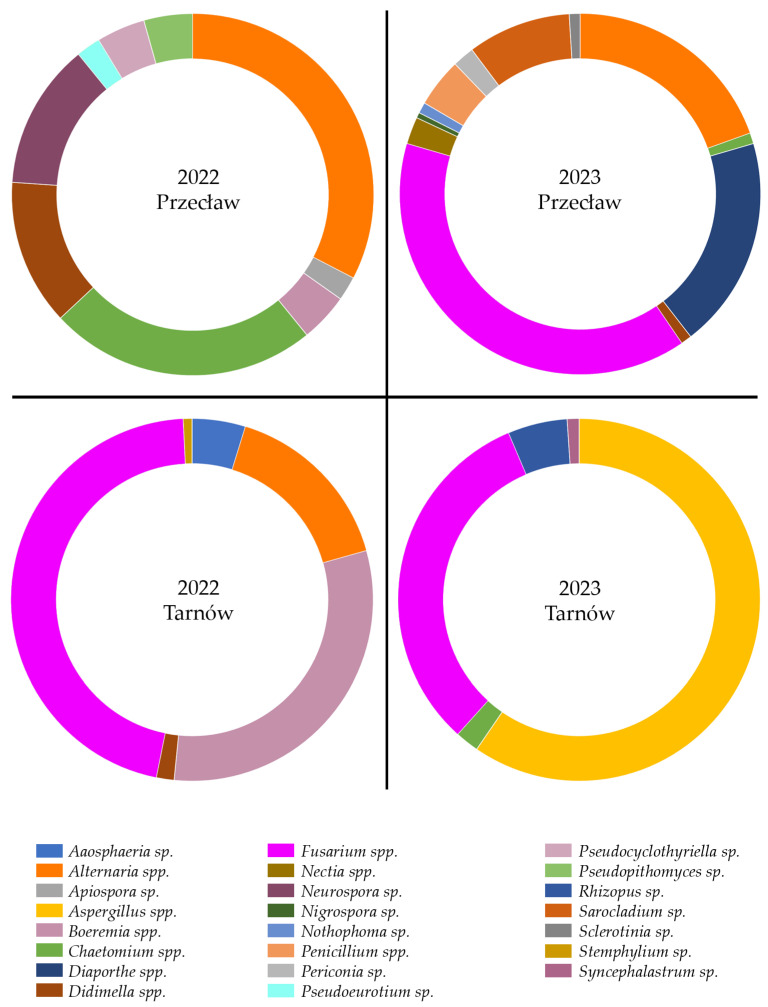
Pie charts illustrating the percentage of dominant fungal genera isolated from soybean seeds collected in Tarnów and Przecław in 2022 and 2023.

In 2022, *Alternaria* spp. and *Chaetomium* spp. species dominated in Przecław, while *Boeremia* spp. and *Fusarium* spp. species prevailed in Tarnów (Figure 2). In 2023, *Fusarium* spp. and *Diaporthe* spp. were the most common in Przecław. In Tarnów, a greater number of *Aspergillus* spp. and *Rhizopus* sp. species were recorded. In all years and locations *Fusarium* spp. and *Alternaria* spp. species were consistently the most frequently occurring genera (Figure 2).

The total number of isolates obtained from each cultivar was as follows: *Cerez PZO*—128 isolates, *Erica*—169 isolates, and *Es Commandor*—174 isolates (taking into account both years and locations). The scale of fungal isolation was comparable among the tested cultivars, and no significant differences were observed between them.

Significant correlations were found between fungal isolates and selected characteristics of soybean seeds (Table 7). The total number of fungal isolates was negatively correlated with total oil content (r = −0.58, *p* < 0.05) and positively correlated with nitrogen content (r = 0.69, *p* < 0.05). The number of *Aspergillus* isolates was also positively correlated with nitrogen content (r = 0.64, *p* < 0.05) (Table 7).

## 4. Discussion

This study demonstrated that the quality of soybean seeds from organic farming, measured as germination capacity, was strongly influenced by environmental conditions. In 2023, a germination decreased significantly compared to 2022 for most cultivars and locations. This decrease may be due to lower temperatures in the early stages of plant development and higher rainfall in June, especially in Przecław. Such conditions slow down plant growth and promote prolonged seed moisture, which encourages increased fungal colonization on the seeds. A similar effect of adverse weather conditions on seed germination and fungal occurrence has been reported previously [17,18,19,20,21,22,43].

Despite lower germination capacity in 2023, the thousand seed weight (TSW) was generally higher than in 2022. This suggests that weather conditions and increased fungal colonization did not cause a reduction in seed weight. This could have been influenced by higher precipitation in 2023, especially in Przecław, where no signs of drought were observed. Instead, increased fungal colonization they mainly affected the chemical parameters and viability of the seeds.

Studies on fungal communities in soybean seeds showed marked differences in abundance and diversity between years. The highest number of isolates was recorded in 2023, especially in Przecław, where precipitation and air humidity were higher. The almost twofold increase in the number of isolates in 2023 confirms the thesis that weather conditions during the growing season play a key role in the development of microbiota [18].

Previous studies comparing organic and conventional farming show clear differences in the species composition and number of fungi [44]. Organic farming promotes greater diversity and abundance of fungi. Conventional farming, based on synthetic products, may reduce the species diversity of microorganisms. Studies of the soybean plant microbiome have shown that the species composition of fungi differed between organic and conventional systems [45]. Although the studies mainly focused on the rhizosphere, the results suggest that fungi associated with seeds may also differ. This may affect seed quality and pathogen pressure.

Therefore, studying fungi present in soybean seeds from farming systems is important for understanding how different agricultural practices shape seed microflora. As few studies have addressed this topic, our research provides new insights.

Many fungal pathogens can infect soybean seeds, causing a variety of symptoms [6]. In this study, most seed samples were colonized by diverse fungi, with a total of 24 genera and 34 species isolated. These results confirm the high diversity of fungi associated with soybean seeds, as previously reported [6,23,24].

In this study, the dominant genus was *Fusarium*, accounting for more than one-third of all isolates, which underscores the importance as a major seed-borne pathogen complex in soybean. The cosmopolitan genus *Fusarium* is widespread and includes many species. Several species, such as *F. acuminatum*, *F. avenaceum*, *F. culmorum*, *F. equiseti*, and *F. oxysporum*, are commonly isolated from soybean seeds and pods. These fungi can reduce seed quality by lowering germination capacity [24,26]. Five *Fusarium* species were identified in this study. *F. equiseti*, *F. culmorum* and *F. flagelliofore* species were the most frequent.

In addition to *Fusarium*, other fungi have also been often isolated from soybean seeds, including *Alternaria*, *Aspergillus* and *Diaporthe* [28,46,47,48]. In this study, *Alternaria* was the second most common genus, while *Aspergillus* ranked third. *Alternaria* is generally considered a facultative parasite of soybean and can occur in up to 95% of freshly harvested seeds [49]. Species of this genus are often linked to seed discoloration and reduced germination capacity [7].

Species of the genus *Aspergillus*, typically regarded as storage fungi, were also found in large numbers in the soybean seeds tested, particularly in 2023. A significant negative correlation was observed between the abundance of *Aspergillus* and the oil content of the seeds. This provides quantitative evidence that fungal colonization reduces the nutritional quality of soybean seeds. Similar effects have been reported previously, as *Aspergillus* species produce lipolytic enzymes that break down lipid reserves. This process leads to a reduction in oil content and overall seed quality [8,50]. Most previous studies focused on qualitative or biochemical changes rather than quantitative relationships between fungal colonization and seed composition [8,29,51]. Our results provide new evidence that higher numbers of *Aspergillus fungi* directly reduce the nutritional value of soybean seeds through lipid degradation.

Overall, this study indicates that unfavorable weather conditions during the growing season promote fungal colonization on soybean seeds. This has a negative effect on germination and nutritional value. Monitoring the dominant fungal taxa and their quantitative impact on seed quality provides valuable information for producing healthy seeds. A high number of fungal isolates correlates with lower germination rates [9]. These results highlight the importance of environmental monitoring and proper seed management to maintain seed quality and ensure pathogen-free planting material. They also provide a basis for further research on improving seed health management and developing strategies to reduce fungal infections in soybean production.

## Figures and Tables

**Table 1:** Soybean cultivars, experimental field locations, and maturity group classification.

No.	Cultivar	Province	Location	Maturity Group *
1	Erica	dolnośląskie	Tarnów	1–2 and 2
2	Es Commandor	dolnośląskie	Tarnów	5–6 and 6
3	Ceres PZO	dolnośląskie	Tarnów	4–5 and 5
4	Erica	podkarpackie	Przecław	1–2 and 2
5	Es Commandor	podkarpackie	Przecław	5–6 and 6
6	Cerez PZO	podkarpackie	Przecław	4–5 and 5

* Soybean maturity group classification based on the COBORU rating scale [34]; 1–2 and 2—very early to early maturity; 4–5 and 5—medium-early maturity; 5–6 and 6—medium-late maturity.

**Table 2:** Mean monthly air temperature at 2 m above the ground and monthly precipitation totals during the 2022 and 2023 growing seasons in Przecław and Tarnów.

Location	Year	Month						
		IV	V	VI	VII	VIII	IX	X
		Temperature [C°]						
Przecław	2022	7.1	14.1	18.3	19.1	20.3	12.5	10.5
Przecław	2023	7.5	12.7	16.8	20.2	20.4	17.3	12.2
Tarnów	2022	6.9	14.3	18.5	18.6	19.5	13.2	12.8
Tarnów	2023	7.0	12.0	17.2	19.9	19.9	17.9	12.3
		**Precipitation [mm]**						
Przecław	2022	62.0	21.1	21.3	84.5	38.0	64.0	32.0
Przecław	2023	45.0	80.0	83.0	107.0	157.0	50.0	77.0
Tarnów	2022	42.2	55.5	74.3	84.1	115.8	80.0	90.0
Tarnów	2023	64.0	41.0	68.0	66.0	135.0	28.0	42.0

**Table 3:** PCR primers used for library preparation.

Primer	Sequence 5′→3′ *	Region	Ref.
NGS_ITS1FI2	TCGTCGGCAGCGTCAGATGTGTATAAGAGACAG GAACCWGCGGARGGATCA	*ITS*	[30,38]
NGS_ITS2	GTCTCGTGGGCTCGGAGATGTGTATAAGAGACAG CGCTGCGTTCTTCATCG		
NGS_EF1	TCGTCGGCAGCGTCAGATGTGTATAAGAGACAG ATGGGTAAGGARGACAAGAC	*TEF*	[39]
NGS_EF2	GTCTCGTGGGCTCGGAGATGTGTATAAGAGACAG GGARGTACCAGTSATCATG		
NGS_5f2	TCGTCGGCAGCGTCAGATGTGTATAAGAGACAG GGGGWGAYCAGAAGAAGGC	*RPB2*	[39]
NGS_7cr	GTCTCGTGGGCTCGGAGATGTGTATAAGAGACAG CCCATRGCTTGYTTRCCCAT		

* Illumina adapter overhang nucleotide sequences (underlined) were added to the gene-specific sequences.

**Table 4:** Mean germination capacity and thousand seed weight (TSW) of three soybean cultivars grown at two locations (Tarnów i Przecław) in 2022 and 2023.

Cultivar	Location	Year of Harvest	Germination [%]	Thousand Seed Weight [g]
Erica	Tarnów	2022	81.67 ± 5.13 ^d *^	195.0
Erica	Tarnów	2023	64.00 ± 5.29 ^c^	222.0
Erica	Przecław	2022	95.67 ± 1.53 ^a^	192.0
Erica	Przecław	2023	68.67 ± 5.13 ^c^	228.0
Es Commandor	Tarnów	2022	97.67 ± 0.58 ^a^	234.4
Es Commandor	Tarnów	2023	43.67 ± 4.16 ^b^	231.0
Es Commandor	Przecław	2022	95.33 ± 1.53 ^a^	204.0
Es Commandor	Przecław	2023	45.67 ± 3.51 ^b^	234.0
Cerez PZO	Tarnów	2022	97.00 ± 1.00 ^a^	231.4
Cerez PZO	Tarnów	2023	43.00 ± 6.50 ^b^	248.0
Cerez PZO	Przecław	2022	99.33 ± 1.55 ^a^	232.0
Cerez PZO	Przecław	2023	95.00 ± 3.51 ^a^	253.0

* Values followed by different small letters are significantly different (Tukey’s HSD, *p* < 0.05).

**Table 5:** Fungal isolates identified from soybean seeds.

No.	Genus	Number of Isolates	Percentage of Isolates
1	*Fusarium* spp.	168	35.7%
2	*Alternaria* spp.	75	15.9%
3	*Aspergillus* spp.	56	11.9%
4	*Boeremia* spp.	41	8.7%
5	*Diaporthe* spp.	39	8.3%
6	*Sarocladium* sp.	19	4.0%
7	*Didymella* spp.	10	2.1%
8	*Penicillium* spp.	9	1.9%
9	*Cladosporium* spp.	9	1.9%
10	*Aaosphaeria* sp.	6	1.3%
11	*Chaetomium* spp.	6	1.3%
12	*Neurospora* sp.	6	1.3%
13	*Rhizopus* sp.	5	1.1%
14	*Nectia* spp.	5	1.1%
15	*Periconia* sp.	4	0.8%
16	*Nothophoma* sp.	2	0.4%
17	*Pseudocyclothyriella* sp.	2	0.4%
18	*Sclerotinia* sp.	2	0.4%
19	*Pseudopithomyces* sp.	2	0.4%
20	*Pseudeurotium* sp.	1	0.2%
21	*Apiospora* sp.	1	0.2%
22	*Nigrospora* sp.	1	0.2%
23	*Stemphylium* sp.	1	0.2%
24	*Syncephalastrum* sp.	1	0.2%
	Total	471	100%

**Table 6:** Frequency of fungal species isolated from seeds of three soybean cultivars grown at two locations (Tarnów and Przecław) during 2022–2023.

Species	Year											
	2022						2023					
	Location											
	Przecław			Tarnów			Przecław			Tarnów		
	Cultivar											
	Cerez PZO	Erica	ES Commandor	Cerez PZO	Erica	ES Commandor	Cerez PZO	Erica	ES Commandor	Cerez PZO	Erica	ES Commandor
*Aaosphaeria arxii* (Aa) Aptroot				6								
*Alternaria alternata* (Fr.) Keissl.							2					
*Alternaria prunicola* Chethana, J.Y. Yan, Xing H. Li & K.D. Hyde	10	4		4	14	2	15	23	2			
*Alternaria* spp.			1									
*Apiospora obovata* (Mei Wang & L. Cai) Pintos & P. Alvarado	1											
*Aspergillus flavus* Link												18
*Aspergillus fumigatus* Fresen										2		
*Aspergillus luchuensis* Inui (dawniej *piperis)*												9
*Aspergillus montevidensis* Talice & J.A. Mackinnon										3		
*Aspergillus pseudoglaucus* Blochwitz										4		
*Aspergillus ruber* (Jos. Konig, Spieck. & W. Bremer) Thom & Church										1		
*Aspergillus tubingensis* Mosseray												19
*Boeremia exigua* (Desm.) Aveskamp, Gruyter & Verkley	2											
*Boeremia* spp.				27		12						
*Chaetomium cervicicola* X. Wei Wang, Crous & L. Lombard		4								2		
*Cladosporium herbarum* (Pers.) Link	4											
*Cladosporium* spp.		3					1		1			
*Diaporthe cotoneastri* (Punith.) Udayanga, Crous & K.D. Hyde							1	17				
*Diaporthe eres* Nitschke dawniej *Diaporthe velata*							5	4	5			
*Diaporthe subclavata* F. Huang, K.D. Hyde & Hong Y. Li							3	4				
*Didymella* spp.	1		5			2	2					
*Fusarium acuminatum* Ellis & Everh					4							
*Fusarium avenaceum* (Fr.) Sacc.					3			2	12			
*Fusarium culmorum* (Wm. G.Sm.) Sacc.					1		6	1			19	
*Fusarium equiseti* (Corda) Sacc.				1	1	10	7	9	26		11	
*Fusarium flagelliforme* J.W. Xia, L. Lombard, Sand.-Den., X.G. Zhang & Crous					23			3				
*Fusarium graminearum* Schwabe				4	1	7						
*Fusarium lateritium* Nees, dawniej *Gibberella baccata*					3		3	1				
*Fusarium sporotrichioides* Sherb.							3		7			
*Nectria* spp.							2	3				
*Neurospora terricola* Goch. & Backus		3	3									
*Nigrospora* sp.							1					
*Nothophoma variabilis* Valenz.- Lopez, Cano, Guarro & Stchigel							2					
*Penicillium brevicompactum* Dierckx									1			
*Penicillium griseofulvum* Dierckx									4			
*Penicillium* spp.								2	2			
*Periconia celtidis* Tennakoon, C.H. Kuo & K.D. Hyde								4				
*Pseudeurotium hygrophilum* (*Sogonov*, *W. Gams*, *Summerb. & Schroers*) *Minnis & D.L. Lindner*	1											
*Pseudocyclothyriella clematidis* (Phukhams. & K.D. Hyde) Phukhams. & Phookamsak		2										
*Pseudopithomyces rosae* Phukhams., Camporesi & K.D. Hyde	2											
*Rhizopus arrhizus* a. Fisch.												5
*Sarocladium strictum* (W. Gams) Summerb.									19			
*Sclerotinia* sp.							2					
*Stemphylium vesicarium* (Wallr.) E.G. Simmons						1						
*Syncephalastrum racemosum* Cohn ex J. Schrot.												1

**Table 7:** Pearson’s correlation coefficients between selected soybean seed traits of soybean and the number of fungal isolates.

Seed Features	Total Oil [%]	Protein [%]	Nitrogen [%]	Moisture [%]	Germination [%]
All fungi	−0.58 *	0.54	0.69 *	0.52	−0.35
*Alternaria* spp.	−0.14	0.14	0.37	0.50	0.25
*Aspergillus* spp.	−0.36	0.43	0.64 *	−0.43	−0.56
*Boaremia* spp.	0.41	−0.44	−0.13	0.18	0.40
*Diaporthe* spp.	−0.50	−0.49	0.41	0.37	−0.11
*Fusarium* spp.	−0.33	0.27	0.11	0.38	−0.25

Only statistically significant correlations are marked with an asterisk (* *p* < 0.05).

## Data Availability

The original contributions presented in this study are included in the article/Supplementary Materials. Further inquiries can be directed to the corresponding author.

## Supplementary Materials

The following supporting information can be downloaded at: https://www.mdpi.com/article/10.3390/pathogens15020239/s1, Table S1: Results of BLASTn analysis of *ITS* region sequences. Table S2: Results of BLASTn analysis of RNA polymerase II beta subunit (*RPB2*) gene and translation elongation factor 1-alpha (*TEF1*) gene sequences.
