# Supplementary material for: Characterization of Fungi Communities in Organic Soybean Seeds Using DNA Sequencing: Effects of Cultivar and Location
Source: Pathogens. 2026 Feb 23;15(2):239. doi: 10.3390/pathogens15020239 (PMC12943531; doi:10.3390/pathogens15020239)
Supplement: Supplementary file 1 [file pathogens-15-00239-s001.zip › Table S1.pdf]

Table S1. Results of BLASTn analysis of ITS region sequences.

| Accession number | Isolate number | Top three results from BLASTn | | | | |
|---|---|---|---|---|---|---|
| | | Accession number | Accession description | Query cover | Percent identity | E-value |
| PX875849 | 1 | PQ741150.1 | *Fusarium sporotrichioides* clone New.CleanUp.Reference OTU7921 | 100% | 99.55% | 6e-108 |
| | | GU253297.1 | *Fusarium sp.* CPCC 480357 18S ribosomal RNA gene | 100% | 99.55% | 2e-107 |
| | | GU116571.1 | *Fusarium armeniacum* isolate H02-781L-5B 18S ribosomal RNA gene | 100% | 99.55% | 2e-107 |
| PX875850 | 3 | OR810431.1 | *Boeremia exigua* isolate 525_ITS4 small subunit ribosomal RNA gene | 100% | 100.00% | 4e-104 |
| | | MK929031.1 | *Boeremia exigua* isolate 15SF2-3 small subunit ribosomal RNA gene | 100% | 100.00% | 4e-104 |
| | | MF039478.1 | *Boeremia exigua* isolate CR 57 small subunit ribosomal RNA gene | 100% | 100.00% | 4e-104 |
| PX875851 | 12 | MT965748.1 | *Stemphylium vesicarium* isolate NTUCC 20-027 small subunit ribosomal RNA gene | 100% | 100.00% | 2e-123 |
| | | MW245001.1 | *Stemphylium ve*sicarium isolate SV099 small subunit ribosomal RNA gene | 100% | 100.00% | 2e-123 |
| | | PQ517252.1 | *Stemphylium vesicarium* isolate SSV30 small subunit ribosomal RNA gene | 100% | 100.00% | 2e-123 |
| PX875852 | 16 | PQ741150.1 | *Fusarium sporotrichioides* clone New.CleanUp.Reference OTU7921 | 100% | 99.55% | 6e-108 |
| | | GU253297.1 | *Fusarium sp.* CPCC 480357 18S ribosomal RNA gene | 100% | 99.55% | 2e-107 |
| | | GU116571.1 | *Fusarium armeniacum* isolate H02-781L-5B 18S ribosomal RNA gene | 100% | 99.55% | 2e-107 |
| PX875853 | 104 | PX622945.1 | *Fusarium equiseti* isolate iso333 small subunit ribosomal RNA gene | 100% | 100.00% | 2e-108 |
| | | PQ345479.1 | *Fusarium equiseti* strain CFZ-11 small subunit ribosomal RNA gene | 100% | 99.54% | 3e-106 |
| | | ON495943.1 | *Fusarium equiseti* strain WL2 small subunit ribosomal RNA gene | 100% | 99.54% | 3e-106 |
| PX875854 | 105 | OR787445.1 | *Alternaria tenuissima* isolate E12 small subunit ribosomal RNA gene | 100% | 100.00% | 6e-118 |
| | | EF364096.1 | *Alternaria tenuissima* 18S ribosomal RNA gene | 100% | 100.00% | 6e-118 |
| | | PQ741038.1 | *Alternaria ten*uissima clone SH1169340.08FU small subunit ribosomal RNA gene | 100% | 100.00% | 6e-118 |
| PX875855 | 106 | MW151080.1 | *Massarina sp.* strain G136_18 small subunit ribosomal RNA gene | 100% | 100.00% | 2e-117 |
| | | OR237680.1 | *Aaosphaeria arxii* isolate B45 small subunit ribosomal RNA gene | 100% | 99.58% | 3e-116 |

| | | | | | | |
|---|---|---|---|---|---|---|
| | | OW984228.1 | *Aaosphaeria arxii* genomic DNA sequence contains 18S rRNA gene, ITS1, 5.8S rRNA gene, ITS2, 28S rRNA gene | 100% | 99.58% | 3e-116 |
| PX875856 | 108 | OR787445.1 | *Alternaria tenuissima* isolate E12 small subunit ribosomal RNA gene | 100% | 100.00% | 6e-118 |
| | | OQ632521.1 | *Alternaria tenuissima* strain UZB-5 small subunit ribosomal RNA gene | 100% | 100.00% | 6e-118 |
| | | EU054406.1 | Fungal endophyte sp. AcapS5 18S ribosomal RNA gene | 100% | 100.00% | 6e-118 |
| PX875857 | 130 | MW812254.1 | *Fusarium equiseti* isolate 34-ITS4-C09 small subunit ribosomal RNA gene | 100% | 100% | 6e-108 |
| | | KJ188664.1 | *Fusarium equiseti* strain DWS23s2 18S ribosomal RNA gene | 100% | 100% | 6e-108 |
| | | PQ242670.1 | *Fusarium equiseti* isolate Fe small subunit ribosomal RNA gene | 100% | 100% | 6e-108 |
| PX875858 | 132 | PX622936.1 | *Fusarium culmorum* isolate iso267 small subunit ribosomal RNA gene | 100% | 100% | 2e-108 |
| | | OR032972.1 | *Fusarium culmorum* isolate SH1242568.08FU small subunit ribosomal RNA gene | 100% | 100% | 2e-108 |
| | | AJ491291.1 | *Fusarium culmorum* 18S rRNA gene, ITS1, 5.8S rRNA gene and ITS2, isolate DAR40014 | 100% | 100% | 6e-108 |
| PX875859 | 139 | MK415057.1 | *Chaetomium globosum* isolate RWL16 small subunit ribosomal RNA gene | 100% | 99.19% | 4e-120 |
| | | MT732805.1 | *Chaetomium sp.* 1 PB-2018a strain 39E small subunit ribosomal RNA gene | 100% | 99.19% | 4e-120 |
| | | AY429056.1 | *Chaetomium globosum* isolate Cg9 18S ribosomal RNA gene | 100% | 99.19% | 4e-120 |
| PX875860 | 150 | OR033018.1 | *Neurospora terricola* iso SH1517597.08FU small subunit ribosomal RNA gene | 100% | 100% | 1e-130 |
| | | KY930619.1 | *Sordaria fimicola* strain C1 18S ribosomal RNA gene | 100% | 99.61% | 5e-129 |
| | | PV454709.1 | *Sordaria fimicola* isolate I-Ge-9 small subunit ribosomal RNA gene | 100% | 99.61% | 5e-129 |
| PX875861 | 151 | OQ248226.1 | *Didymella pinodella* strain ColD Pav_26 small subunit ribosomal RNA gene | 100% | 100% | 4e-104 |
| | | PP947354.1 | *Epicoccum plurivorum* strain CN103E2 small subunit ribosomal RNA gene | 100% | 100% | 4e-104 |
| | | PP947345.1 | *Epicoccum plurivorum* strain CN056A5 small subunit ribosomal RNA gene | 100% | 100% | 4e-104 |
| PX875862 | 171 | HM222950.1 | *Arthrinium phaeospermum* isolate A218 18S ribosomal RNA gene | 100% | 99.64% | 1e-140 |
| | | JX321747.1 | Uncultured fungus clone 034A4061 18S ribosomal RNA gene | 100% | 99.29% | 6e-139 |
| | | MK560165.1 | *Arthrinium phaeospermum* isolate DAFE_SP16-4 small subunit ribosomal RNA gene | 100% | 99.29% | 6e-139 |
| PX875863 | 172 | MT529748.1 | *Pseudeurotium hygrophilum* clone SF_472 small subunit ribosomal RNA gene | 100% | 100% | 3e-116 |

| | | | | | | |
|---|---|---|---|---|---|---|
| | | MH857368.1 | *Pseudeurotium ovale* culture CBS:389.54 strain CBS 389.54 small subunit ribosomal RNA gene | 100% | 100% | 3e-116 |
| | | PV661786.1 | *Pseudeurotium sp.* strain CBI-S032 small subunit ribosomal RNA gene | 100% | 100% | 3e-116 |
| PX875864 | 173 | L25431.1 | *Cladosporium herbarum* ribosomal RNA gene fragment | 100% | 100% | 2e-112 |
| | | PQ741214.1 | *Cladosporium herbarum* clone SH1572792.08FU small subunit ribosomal RNA gene | 100% | 100% | 2e-112 |
| | | KC765990.1 | Uncultured fungus clone 25F26 18S ribosomal RNA gene | 100% | 100% | 2e-112 |
| PX875865 | 176 | OR691764.1 | *Pseudopithomyces chartarum* strain ICMP 12591 small subunit ribosomal RNA gene | 100% | 100% | 4e-140 |
| | | OR691724.1 | *Pseudopithomyces chartarum* strain ICMP 1276 small subunit ribosomal RNA gene | 100% | 100% | 4e-140 |
| | | AM902015.1 | Uncultured ascomycete ITS region including 18S rRNA gene, ITS1, 5.8S rRNA gene, ITS2 and 28S rRNA gene | 100% | 100% | 4e-140 |
| PX875866 | 177 | KF038324.1 | *Boeremia exigua var. exigua* culture-collection ICMP:19750 18S ribosomal RNA gene | 100% | 100% | 4e-104 |
| | | ON599115.1 | *Boeremia exigua* isolate BLYP136B_2018 small subunit ribosomal RNA gene | 100% | 100% | 4e-104 |
| | | MH859059.1 | *Boeremia exigua var. exigua* culture CBS:596.67 strain CBS 596.67 small subunit ribosomal RNA gene | 100% | 100% | 4e-104 |
| PX875867 | 201 | MG589146.1 | *Cladosporium phaenocomae* strain HU9278 small subunit ribosomal RNA gene | 100% | 100% | 8e-113 |
| | | MN518420.1 | *Cladosporium sp.* strain GPS3-1 small subunit ribosomal RNA gene | 100% | 100% | 8e-113 |
| | | KC525562.1 | Uncultured *Cladosporium* clone MDL1-6501 18S ribosomal RNA gene | 100% | 100% | 8e-113 |
| PX875868 | 202 | EU520640.1 | Uncultured ascomycete clone W8 internal transcribed spacer 1 | 100% | 100% | 4e-120 |
| | | KF977132.1 | Uncultured fungus clone K27 18S ribosomal RNA gene | 100% | 99.59% | 2e-118 |
| | | PV608521.1 | *Pseudocyclothyriella sp.* YG-2025b strain QG18A isolate CGMCC 3.24509 small subunit ribosomal RNA gene | 100% | 98.76% | 4e-115 |
| PX875869 | 258 | KT192371.1 | *Nigrospora oryzae* strain 1-TOP-2 18S ribosomal RNA gene | 100% | 99.55% | 1e-109 |
| | | KF227809.1 | *Nigrospora sp.* BSL2_5 18S ribosomal RNA gene | 100% | 99.55% | 1e-109 |
| | | MN341501.1 | *Nigrospora sp.* isolate KoRLI046192 small subunit ribosomal RNA gene | 100% | 99.11% | 6e-108 |
| PX875870 | 260 | PX622943.1 | *Diaporthe subclavata* isolate iso328 small subunit ribosomal RNA gene | 100% | 100% | 6e-123 |
| | | MT613812.1 | *Diaporthe sp.* isolate T.47 small subunit ribosomal RNA gene | 100% | 99.59% | 3e-121 |
| | | MT613751.1 | *Diaporthe sp.* isolate G.19 small subunit ribosomal RNA gene | 100% | 99.59% | 3e-121 |

| | | | | | | |
|---|---|---|---|---|---|---|
| PX875872 | 263 | PX622934.1 | *Diaporthe eres* isolate iso265 small subunit ribosomal RNA gene | 100% | 100% | 2e-124 |
| | | KJ160576.1 | *Diaporthe eres* strain CPC 23806 18S ribosomal RNA gene | 100% | 99.60% | 8e-123 |
| | | MT613852.1 | *Diaporthe sp.* isolate T.87 small subunit ribosomal RNA gene | 100% | 99.60% | 8e-123 |
| PX875873 | 268 | JN032733.1 | *Diaporthe melonis* isolate DB260511 18S ribosomal RNA gene | 100% | 99.60% | 7e-123 |
| | | MH137752.1 | *Diaporthe cucurbitae* isolate M_FA_L8 small subunit ribosomal RNA gene | 100% | 99.60% | 7e-123 |
| | | KC916708.1 | *Phomopsis vaccinii* voucher TC2-014 18S ribosomal RNA gene | 100% | 99.60% | 7e-123 |
| PX875874 | 283 | KF859918.1 | *Botrytis cinerea* culture-collection CBS:131.28 18S ribosomal RNA gene | 100% | 100% | 8e-108 |
| | | PP781115.1 | *Botrytis fabae* strain MG-7 small subunit ribosomal RNA gene | 100% | 100% | 8e-108 |
| | | MH208266.1 | *Botrytis cinerea* isolate ASF-Bc1 small subunit ribosomal RNA gene | 100% | 100% | 8e-108 |
| PX875875 | 284 | PV474681.1 | *Fusarium oxysporum* isolate ITS-MA112 small subunit ribosomal RNA gene | 100% | 100% | 6e-109 |
| | | AJ491296.1 | *Fusarium avenaceum* 18S rRNA gene, 5.8S rRNA gene, ITS1 and ITS2, isolate DAR49137 | 100% | 100% | 6e-109 |
| | | FJ602986.1 | *Gibberella avenacea* isolate FA40 18S ribosomal RNA gene | 100% | 100% | 6e-108 |
| PX875876 | 287 | OM965333.1 | *Didymella microchlamydospora* isolate AB17M_11 small subunit ribosomal RNA gene | 100% | 100% | 2e-103 |
| | | MT420629.1 | *Phoma sp.* isolate R30 small subunit ribosomal RNA gene | 100% | 100% | 2e-103 |
| | | OR500019.1 | *Didymella dimorpha* strain CN067H2 small subunit ribosomal RNA gene | 100% | 100% | 2e-103 |
| PX875877 | 297 | OQ255930.1 | *Penicillium griseofulvum* isolate TS2 small subunit ribosomal RNA gene | 100% | 100% | 2e-123 |
| | | GQ999396.1 | Uncultured fungus clone LX042233-122-012-B09 internal transcribed spacer 1 | 100% | 100% | 2e-123 |
| | | JF429675.1 | *Penicillium sp.* EMA-2011b 18S ribosomal RNA gene | 100% | 100% | 2e-123 |
| PX875878 | 299 | PX622940.1 | *Sarocladium strictum* isolate iso298 small subunit ribosomal RNA gene | 100% | 100% | 2e-118 |
| | | AY138845.1 | *Sarocladium strictum* genogroup I strain CBS 346.70T 18S ribosomal RNA gene | 100% | 99.58% | 1e-116 |
| | | OP970583.1 | *Sarocladium sp.* strain Bail6 small subunit ribosomal RNA gene | 100% | 99.58% | 1e-116 |
| PX875879 | 300 | OQ255930.1 | *Penicillium griseofulvum* isolate TS2 small subunit ribosomal RNA gene | 100% | 100% | 2e-123 |
| | | GQ999396.1 | Uncultured fungus clone LX042233-122-012-B09 internal transcribed spacer 1 | 100% | 100% | 2e-123 |

| | | | | | | |
|---|---|---|---|---|---|---|
| | | JF429675.1 | *Penicillium sp.* EMA-2011b 18S ribosomal RNA gene, partial sequence | 100% | 100% | 2e-123 |
| PX875880 | 331 | PX622944.1 | *Periconia celtidis* isolate iso331 internal transcribed spacer 1 and 5.8S ribosomal RNA gene | 100% | 100% | 1e-125 |
| | | OP058997.1 | *Periconia byssoides* voucher 202105053 small subunit ribosomal RNA gene | 100% | 99.60% | 5e-124 |
| | | OQ257177.1 | *Periconia byssoides* isolate E343 small subunit ribosomal RNA gene | 100% | 99.60% | 5e-124 |
| PX875881 | 338 | OM265285.1 | *Aspergillus flavus* isolate Manjari Shyama (MS5) small subunit ribosomal RNA gene | 100% | 100% | 3e-127 |
| | | MW314793.1 | *Aspergillus flavus* isolate Asp_f_1 small subunit ribosomal RNA gene | 100% | 100% | 3e-127 |
| | | MT584825.1 | *Aspergillus flavus* isolate BB-1 small subunit ribosomal RNA gene | 100% | 100% | 3e-127 |
| PX875882 | 339 | LC573598.1 | *Aspergillus luchuensis* NBRC:4314 genes for 18S rRNA, ITS1, 5.8S rRNA, ITS2 and 28S rRNA | 100% | 100% | 2e-129 |
| | | MH856956.1 | *Aspergillus luchuensis* culture CBS:128.52 strain CBS 128.52 small subunit ribosomal RNA gene | 100% | 100% | 2e-129 |
| | | OQ826679.1 | *Aspergillus piperis* isolate BLS11 small subunit ribosomal RNA gene | 100% | 100% | 2e-129 |
| PX875883 | 340 | MG659678.1 | *Aspergillus tubingensis* strain ND84 small subunit ribosomal RNA gene | 100% | 100% | 2e-129 |
| | | OR298260.1 | *Aspergillus niger* isolate Y02 small subunit ribosomal RNA gene | 100% | 100% | 2e-129 |
| | | PV108456.1 | *Aspergillus phoenicis* strain CIRM-BRFM 1500 ITS 1 | 100% | 100% | 2e-129 |
| PX875884 | 343 | PX622947.1 | *Syncephalastrum racemosum* isolate iso343 small subunit ribosomal RNA gene | 100% | 100% | 7e-143 |
| | | OQ734577.1 | *Syncephalastrum racemosum* isolate 3 small subunit ribosomal RNA gene | 100% | 99.29% | 6e-139 |
| | | KT336543.1 | *Syncephalastrum sp.* M39 18S ribosomal RNA gene, partial sequence | 100% | 99.29% | 6e-139 |
| PX875885 | 346 | MF044049.1 | *Aspergillus pseudoglaucus* strain FJAT-31014 small subunit ribosomal RNA gene | 100% | 100% | 1e-106 |
| | | OP596008.1 | *Aspergillus pseudoglaucus* strain CN091E4 small subunit ribosomal RNA gene | 100% | 100% | 1e-106 |
| | | MN454494.1 | *Aspergillus glaucus* isolate 003 small subunit ribosomal RNA gene | 100% | 100% | 1e-105 |
| PX875886 | 348 | KP068712.1 | *Aspergillus fumigatus* strain WM 06.611 isolate ISHAM-ITS_ID MITS2806 18S ribosomal RNA gene | 100% | 100% | 6e-129 |
| | | OP379476.1 | *Aspergillus fumigatus* isolate ZNF1 small subunit ribosomal RNA gene | 100% | 100% | 6e-129 |
| | | PQ876437.1 | *Aspergillus sp.* isolate P0-018 small subunit ribosomal RNA gene | 100% | 100% | 6e-129 |
| PX875887 | 349 | PQ683349.1 | *Aspergillus montevidensis* isolate 15_ITS4_A09_3730XL small subunit ribosomal RNA gene | 100% | 100% | 1e-105 |

| | | | | | | |
|---|---|---|---|---|---|---|
| | | MW879114.1 | *Aspergillus montevidensis* strain EXF-7594 small subunit ribosomal RNA gene | 100% | 100% | 1e-105 |
| | | OP596041.1 | *Aspergillus chevalieri* strain CN096H8 small subunit ribosomal RNA gene | 100% | 100% | 1e-105 |
| PX875888 | 354 | OW983603.1 | *Penicillium brevicompactum* genomic DNA sequence contains 18S rRNA gene, ITS1, 5.8S rRNA gene, ITS2, 28S rRNA gene | 100% | 100% | 8e-123 |
| | | OR808072.1 | *Penicillium brevicompactum* isolate 31c_6 small subunit ribosomal RNA gene | 100% | 100% | 8e-123 |
| | | OP315774.1 | *Penicillium brevicompactum* isolate PL096 small subunit ribosomal RNA gene | 100% | 100% | 8e-123 |
| PX875889 | 356 | PX622952.1 | *Rhizopus arrhizus* isolate iso356 small subunit ribosomal RNA gene | 100% | 100% | 1e-166 |
| | | MK988569.1 | *Rhizopus arrhizus* isolate AB313 small subunit ribosomal RNA gene | 100% | 100% | 4e-166 |
| | | PP502936.1 | *Rhizopus arrhizus* isolate Sun_ITS01_Kz small subunit ribosomal RNA gene | 100% | 100% | 4e-166 |
| PX875890 | 359 | OQ931146.1 | *Alternaria alternata* isolate vvunid16yl16 small subunit ribosomal RNA gene | 100% | 100% | 8e-118 |
| | | KU258751.1 | *Alternaria alternata* isolate HT-R-24 18S ribosomal RNA gene | 100% | 100% | 8e-118 |
| | | MT451845.1 | *Alternaria alternata* strain AA-3 small subunit ribosomal RNA gene | 100% | 100% | 8e-118 |
| PX875891 | 368 | MH855375.1 | *Aspergillus ruber* culture CBS:106.33 strain CBS 106.33 small subunit ribosomal RNA gene | 100% | 100% | 1e-105 |
| | | OW982555.1 | *Aspergillus ruber* genomic DNA sequence contains 18S rRNA gene, ITS1, 5.8S rRNA gene, ITS2, 28S rRNA gene | 100% | 100% | 1e-105 |
| | | PP921317.1 | *Aspergillus niveoglaucus* isolate FIS7 internal transcribed spacer 1, partial sequence; 5.8S ribosomal RNA gene | 100% | 100% | 1e-105 |
