# Supplementary material for: Characterization of Fungi Communities in Organic Soybean Seeds Using DNA Sequencing: Effects of Cultivar and Location
Source: Pathogens. 2026 Feb 23;15(2):239. doi: 10.3390/pathogens15020239 (PMC12943531; doi:10.3390/pathogens15020239)
Supplement: Supplementary file 1 [file pathogens-15-00239-s001.zip › Table S2.pdf]

Table S2. Results of BLASTn analysis of RNA polymerase II beta subunit (RPB2) gene and translation elongation factor 1-alpha (TEF1) gene sequences.

Accession number	Isolate number	Top three results from BLASTn				
		**Accession number**	**Accession description**	**Query cover**	**Percent identity**	**E-value**
PZ020375[RPB2]	1	MN183573.1	*Fusarium sporotrichioides* isolate NY17-6-207-13 RNA polymerase II beta subunit (RPB2) gene	100%	100%	9e-106
		MN183652.1	*Fusarium sporotrichioides* isolate NY17-8-201-11 RNA polymerase II beta subunit (RPB2) gene	100%	100%	9e-106
		MH582097.1	*Fusarium sporotrichioides* strain MRC 1708 RNA polymerase II (RPB2) gene	100%	100%	9e-106
PZ020383[TEF1]	1	PP833608.1	*Fusarium sporotrichioides* isolate xjau408-4 translation elongation factor 1-alpha (tef1) gene	100%	100%	2e-102
		MZ078870.1	*Fusarium sporotrichioides* strain 495E translation elongation factor 1-alpha (tef1) gene	100%	100%	2e-102
		PP098295.1	*Fusarium sporotrichioides* isolate 23KaPT10_6 translation elongation factor 1-alpha (TEF1) gene	100%	100%	2e-102
PZ020372[RPB2]	16	PQ248117.1	*Fusarium graminearum* strain WV22F11 RNA polymerase II second largest subunit (RPB2) gene	100%	100%	6e-128
		OP785266.1	*Fusarium graminearum* isolate ZL3-2 RNA polymerase II second largest subunit (RPB2) gene	100%	100%	6e-128
		OP785265.1	*Fusarium graminearum* isolate YLY2-1 RNA polymerase II second largest subunit (RPB2) gene	100%	100%	6e-128
PZ020380[TEF1]	16	PV013465.1	*Fusarium graminearum* strain IL2C11I1 translation elongation factor 1-alpha (TEF1a) gene	100%	100%	4e-109
		PV013452.1	*Fusarium graminearum* strain IL3C4I1 translation elongation factor 1-alpha (TEF1a) gene	100%	100%	4e-109
		PV013450.1	*Fusarium graminearum* strain IL3C4I2 translation elongation factor 1-alpha (TEF1a) gene	100%	100%	4e-109
PZ020370[RPB2]	104	PP118284.1	*Fusarium equiseti* isolate HSRF2 RNA polymerase II second largest subunit (RPB2) gene	100%	99.55%	5e-108
		OR727720.1	*Fusarium equiseti* strain MFG 70109 RNA polymerase II second largest subunit (rpb2) gene	100%	99.55%	5e-108
		OR727724.1	*Fusarium equiseti* strain MFG 70168 RNA polymerase II second largest subunit (rpb2) gene	100%	99.55%	5e-108
PZ020378[TEF1]	104	JF966245.1	*Fusarium equiseti* isolate KF2656 translation elongation factor EF-1 gene	100%	99.08%	4e-104
		PV247765.1	*Fusarium equiseti* voucher XWQ6B1 translation elongation factor 1-alpha gene	100%	99.08%	4e-104
		KP400714.1	*Fusarium equiseti* isolate Z331 translation elongation factor 1-alpha (TEF1) gene	100%	99.08%	4e-104
PZ020369[RPB2]	130	PQ459039.1	*Fusarium flagelliforme* strain MFG 70562 RNA polymerase II second largest subunit (rpb2) gene	98%	100%	4e-109
		PQ337250.1	*Fusarium flagelliforme* isolate QJ18122 RNA polymerase II second largest subunit (RPB2) gene	98%	100%	4e-109

		PQ337249.1	*Fusarium flagelliforme* isolate QJ16632 RNA polymerase II second largest subunit (RPB2) gene	98%	100%	4e-109
PZ020377[TEF1]	130	MZ921840.1	*Fusarium flagelliforme* strain NL19-047004 translation elongation factor 1-alpha (tef1) gene	100%	99.53%	4e-104
		GQ505589.1	*Fusarium flagelliforme* strain NRRL 6548 translation elongation factor 1 (EF1) gene	100%	99.53%	4e-104
		PQ325565.1	*Fusarium flagelliforme* isolate QJ2093 translation elongation factor 1-alpha (TEF1) gene	100%	99.53%	4e-104
PZ020371[RPB2]	132	MK848688.1	*Fusarium culmorum* strain QHU2018001 RNA polymerase II (RPB2) gene	100%	100%	2e-107
		PV654535.1	*Fusarium culmorum* strain Karbala-1 RNA polymerase II second largest subunit (RPB2) gene	100%	100%	2e-107
		PV595248.1	*Fusarium culmorum* isolate P612B RNA polymerase II second largest subunit (RPB2) gene	100%	100%	2e-107
PZ020379[TEF1]	132	KP065682.1	*Fusarium culmorum* strain WF19 translation elongation factor 1-alpha gene	100%	100%	5-108
		PV867328.1	*Fusarium culmorum* translation elongation factor 1-alpha (TEF1) gene	100%	100%	5e-108
		KP065683.1	*Fusarium culmorum* strain WF10 translation elongation factor 1-alpha gene	100%	99.54%	2e-106
PZ020373[RPB2]	284	OQ281348.1	*Fusarium lateritium* isolate SICAUCC 22-0138 RNA polymerase II second largest subunit (rpb2) gene	100%	100%	7e-107
		PX130475.1	*Fusarium lateritium* isolate ZJUE1503 RNA polymerase II (RPB2) gene	100%	100%	7e-107
		PX130474.1	*Fusarium lateritium* isolate ZJUE1499 RNA polymerase II (RPB2) gene	100%	100%	7e-107
PZ020381[TEF1]	284	PP921795.1	*Fusarium lateritium* isolate F10R10 translation elongation factor 1-alpha (TEF1) gene	100%	100%	2e-106
		AY707173.1	*Fusarium lateritium* Clade III strain L-55 translation elongation factor 1-alpha gene	100%	100%	2e-106
		JF740854.1	*Fusarium lateritium* strain NRRL 52786 translation elongation factor alpha gene	100%	100%	2e-106
PZ020374[RPB2]	280	PQ248116.1	*Fusarium acuminatum* strain WV22F8 RNA polymerase II second largest subunit (RPB2) gene	100%	100%	2e-107
		PQ201141.1	*Fusarium acuminatum* strain WV22F3 RNA polymerase II second largest subunit (RPB2) gene	100%	100%	2e-107
		PQ201162.1	*Fusarium acuminatum* strain WV22F34 RNA polymerase II second largest subunit (RPB2) gene	100%	100%	2e-107
PZ020382[TEF1]	280	OR440987.1	*Fusarium acuminatum* isolate GA19C11.3I translation elongation factor 1-alpha (TEF1) gene	100%	99.53%	4e-104
		OM687119.1	*Fusarium acuminatum* strain G21YY2-15 translation elongation factor 1-alpha gene	100%	99.53%	4e-104
		PV906195.1	*Fusarium acuminatum* strain G19TH22-7 translation elongation factor 1-alpha (TEF) gene	100%	99.53%	4e-104
PZ020368[RPB2]	288	MK185026.1	*Fusarium avenaceum* isolate YNSF16-66 DNA-directed RNA polymerase II beta subunit (RBP2) gene	100%	100%	4e-109

		PV260667.1	*Fusarium avenaceum* voucher XWQ6H1 RNA polymerase II second largest subunit gene	100%	100%	4e-109
		PQ778786.1	*Fusarium avenaceum* strain XZ-22-9 RNA polymerase II second largest subunit (RPB2) gene	100%	100%	4e-109
PZ020376^{TEF1}	288	PQ563139.1	*Fusarium avenaceum* isolate 180MY translation elongation factor 1-alpha (EF1a) gene	100%	100%	3e-105
		MZ078978.1	*Fusarium avenaceum* strain 43HS translation elongation factor 1-alpha (tef1) gene	100%	100%	3e-105
		JN167211.1	*Fusarium avenaceum* isolate F49 translation elongation factor 1 alpha (EF-1alpha) gene	100%	100%	3e-105
